# Biomarkers of Prognosis and Efficacy of Anti-angiogenic Therapy in Metastatic Clear Cell Renal Cancer

**DOI:** 10.3389/fonc.2019.01400

**Published:** 2019-12-11

**Authors:** Carmine D'Aniello, Massimiliano Berretta, Carla Cavaliere, Sabrina Rossetti, Bianca Arianna Facchini, Gelsomina Iovane, Giovanna Mollo, Mariagrazia Capasso, Chiara Della Pepa, Laura Pesce, Davide D'Errico, Carlo Buonerba, Giuseppe Di Lorenzo, Salvatore Pisconti, Ferdinando De Vita, Gaetano Facchini

**Affiliations:** ^1^Division of Medical Oncology, A.O.R.N. dei COLLI “Ospedali Monaldi-Cotugno-CTO,” Naples, Italy; ^2^Division of Medical Oncology, Istituto Nazionale Tumori, IRCCS CRO Aviano (PN), Milan, Italy; ^3^UOC of Medical Oncology, ASL NA 3 SUD, Ospedali Riuniti Area Nolana, Nola, Italy; ^4^Departmental Unit of Experimental Uro-Andrologic Clinical Oncology, Istituto Nazionale Tumori Fondazione G. Pascale—IRCCS, Naples, Italy; ^5^Division of Medical Oncology, Department of Precision Medicine, University of Campania 'Luigi Vanvitelli', Naples, Italy; ^6^Oncology Unit, San Luca Hospital, Vallo Della Lucania, Italy; ^7^CRTR Rare Tumors Reference Center, AOU Federico II, Naples, Italy; ^8^Environment & Health Operational Unit, Zoo-Prophylactic Institute of Southern Italy, Portici, Italy; ^9^Department of Clinical Medicine and Surgery, University Federico II, Naples, Italy; ^10^Department of Medicine, University of Molise, Campobasso, Italy; ^11^Department of Onco-Hematology, Medical Oncology, S.G. Moscati Hospital, Taranto, Italy

**Keywords:** biomarkers, angiogenesis, immunotherapy, prognosis, renal cell carcinoma, targeted therapy

## Abstract

In the last decades, the prognosis of metastatic renal cell carcinoma (mRCC) has remarkably improved following the advent of the “targeted therapy” era. The expanding knowledge on the prominent role played by angiogenesis in RCC pathogenesis has led to approval of multiple anti-angiogenic agents such as sunitinib, pazopanib, axitinib, cabozantinib, sorafenib, and bevacizumab. These agents can induce radiological responses and delay cancer progression for months or years before onset of resistance, with a clinically meaningful activity. The need for markers of prognosis and efficacy of anti-angiogenic agents has become more compelling as novel systemic immunotherapy agents have also been approved in RCC and can be administered as an alternative to angiogenesis inhibitors. Anti PD-1 monoclonal antibody nivolumab has been approved in the second-line setting after tyrosine kinase inhibitors failure, while combination of nivolumab plus anti CTLA-4 monoclonal antibody ipilimumab has been approved as first-line therapy of RCC patients at intermediate or poor prognosis. In this review article, biomarkers of prognosis and efficacy of antiangiogenic therapies are summarized with a focus on those that have the potential to affect treatment decision-making in RCC. Biomarkers predictive of toxicity of anti-angiogenic agents have also been discussed.

## Introduction

Renal cell carcinoma (RCC) is the seventh most frequently diagnosed malignancy in men and the ninth in women, representing ~2–3% of cancer diagnoses in adults (1). RCC includes a group of tumors with heterogenic features in terms of genetic landscape, growth pattern, and metastatic potential (2). Clear cell RCC (ccRCC) is the most frequently diagnosed histotype (75–80%), followed by papillary RCC (10–15%), chromophobe RCC (5%), collecting duct/medullary carcinomas (1–2%), and translocation-associated RCC (<1%), plus ~5% of unclassified cases (3). The central role played by angiogenesis in RCC pathogenesis is mediated by signaling cascades involving multiple factors, such as pVHL (von Hippel-Lindau tumor suppressor protein), HIF-1α (hypoxia inducible factor 1 subunit alpha), VEGF (vascular endothelial growth factor), PDGF (platelet-derived growth factor), and mTOR (mammalian target of rapamycin) (4–7). At the present time, approved targeted therapy agents in advanced RCC include bevacizumab, a monoclonal antibody that blocks VEGF-A by preventing its binding to the VEGF receptor; tyrosine kinase inhibitors (TKIs) that mainly exert their activity by inhibiting the VEGF receptor and include sorafenib, sunitinib, pazopanib, and axitinib; inhibitors of the mTOR complex, such as temsirolimus and everolimus. The increased RCC risk in immunocompromised patients, the abundance of tumor-infiltrating lymphocytes, as well as the anecdotal reports of spontaneous tumor regressions provide evidence supporting the potential effectiveness of immunotherapy in RCC. Before the advent of the TKIs era, interferon-alpha (IFN-α) and high-dose interleukin-2 (HD IL-2) were among the few active systemic therapeutic options, although sustained responses were obtained only in a small fraction of patients with mRCC (8–13). An exciting breakthrough in the development of immunotherapy of RCC has been achieved with agents directed against molecules that act as critical regulators of tumor-induced immune suppression, such as nivolumab, directed against PD-1 (programmed death-1), and ipilimumab directed against CTLA-4 (Cytotoxic T-Lymphocyte Antigen 4).

This review provides a comprehensive appraisal of prognostic and predictive factors tested in patients with ccRCC treated with anti-angiogenic agents, with a focus on those with a potential to affect therapeutic decisions—for example, the choice of an anti-angiogenic vs. an immunotherapy agent. Biomarkers predictive of toxicity of anti-angiogenic agents have also been presented and discussed.

## Prognostic and Predictive Biomarkers

### Clinical and Biochemistry Markers

Several prognostic models have been developed over the past years (14–16). The Memorial Sloan-Kettering Cancer Center (MSKCC) classification system (17–19) was developed during the cytokine era. Subsequently, with the advent of targeted therapies (TTs), Heng et al. (20, 21) validated the International Metastatic RCC Database Consortium (IMDC) risk score. The MSKCC or Motzer criteria classify patients according to three serum factors (lactate dehydrogenase-LDH, hemoglobin, and calcium) and two clinical factors (Karnofsky performance status and interval from diagnosis to treatment). These variables correlate significantly with overall survival (OS) (Table 1). The IMDC model incorporates six factors, which include two clinical factors (Karnofsky performance status and time from initial RCC diagnosis to the start of therapy), and four serum markers (hemoglobin, calcium, neutrophils, and platelets). In this model, pre-treatment factors independently associated with a shorter OS by multivariate analysis are hemoglobin < lower limit of normal (LLN) (*p* < 0.0001), corrected calcium > ULN (upper limit of normal) (*p* = 0.0006), Karnofsky performance status <80% (*p* < 0.0001), time from diagnosis to treatment <1 year (*p* = 0.01), neutrophils > ULN (*p* < 0.0001), and platelets > ULN (*p* = 0.01). Based on these factors, different overall survivals were reported in the favorable-risk group (no prognostic factors, *n* = 133, median OS = 43.2 months); intermediate-risk group (1–2 prognostic factors, *n* = 301, median OS = 22.5 months); and poor-risk group (3–6 prognostic factors, *n* = 152, median OS = 7.8 months) (Table 2). The importance of such a prognostic classification lies in its implications for treatment choice, as temsirolimus is approved only in patients at poor prognosis and novel immunotherapy combination ipilimumab plus nivolumab is approved in patients at intermediate and poor prognosis (22–31).

**Table 1:** MSKCC score system.

**KARNOFSKY PS**	**<80%**			
Hemoglobin	<lower normal limit			
Lactate dehydrogenase	1.5 × upper normal limit			
Corrected serum calcium	>10 mg/dL			
Period from diagnosis to treatment	<1 year			
**Prognosis**	**Score**	**Median overall survival (months)**	**Survival at 3 years (%)**
Good	0	30	45
Intermediate	1–2	14	17
Poor	3–5	5	2

*MSKCC, Memorial Sloan Kattering Cancer Center*.

**Table 2:** IMDC risk group classification.

**KARNOFSKY PS**	**<80%**			
Hemoglobin	<lower normal limit			
Corrected serum calcium	>10 mg/dL			
Period from diagnosis to treatment	<1 year			
Neutrophil counts	>upper normal limit			
Platelet count	>upper normal limit			
**Prognosis**	**Score**	**Median overall survival (months)**	**Survival at 2 years (%)**
Good	0	NR	75
Intermediate	1–2	27 mos.	53
Poor	3–6	8.8 mos.	7

*IMDC, International Metastatic RCC Database Consortium*.

### Histology Biomarkers

Several histology factors predictive of RCC recurrence have been identified, including pathologic stage, nuclear Fuhrman grade, histology variant, sarcomatoid differentiation or necrosis, vascular invasion, and invasion of the collector system. The nuclear Fuhrman grade is the only histology marker that proved to be independently associated with OS in RCC (15, 16). Tumor stage and grade show prognostic value in most multivariate models (32).One retrospective pathology study showed that more than fifty per cent of the revised clear cell RCC samples had distinctive features from those associated with classical clear cell carcinoma, which suggests that clear cell RCC should be further divided in additional classes (33–37). Of note, RCC patients with a higher clear cell component are more likely to benefit most from VEGF-TT (38). Importantly, different histologies, such as clear cell, papillary, and chromophobe RCCs as well as benign oncocytomas have a different genetic background (39).

Carbonic anhydrase IX (CAIX) is a protein that converts carbon dioxide into carbonic acid, which is essential for pH homeostasis in hypoxic conditions. CAIX can be assessed at immunohistochemistry and is induced by hypoxia. Higher CAIX expression levels predict longer survival in localized RCC and mRCC and have been associated with response to sorafenib treatment (40). C-X-C chemokine receptor type 4 (CXCR4) is another promising biomarker, which may cross-talk with the VEGF pathways. D'Alterio et al. (41) included 62 mccRCC patients treated with first-line sunitinib and evaluated CXCR4 expression at immunohistochemistry. An association between high CXCR4 expression and poor response to sunitinib was detected. Another study revealed that low or no CXCR4 expression in patients receiving sorafenib was associated with higher PFS (20.0 ± 5.9 months) than intermediate or high CXCR4 (6.0 ± 0.8 months) (*p* = 0.038) expression; however, no correlation was found between low or no CXCR4 expression and OS (42).

Higher levels of HIF-1α or HIF-2α at immunohistochemistry correlated with complete or a partial response to sunitinib therapy; particularly high levels of HIF-1α at baseline was associated with longer PFS (42.0 weeks, 95% CI 31.0–56.3) than low HIF-1α levels (30.4 weeks, 95% CI 22.2–43.9, HR = 1.55, *p* = 0.034) (43). Combined immunohistochemistry analysis showed no statistically significant associations between time-to-progression or OS and either HIF-1α or CAIX tumor expression. Nevertheless, PFS was significantly different between HIF-1α-low groups 0–2 (i.e., 0–50%) and HIF-1α-high groups 3–4 (i.e., 51–100%). The same results were obtained in another study in which sunitinib-treated patients reached a significantly longer PFS in the lower HIF-1α (44).

### Serum Biomarkers

Angiogenesis is implicated in RCC tumorigenesis with a multiple involved factor, including VHL, HIF-1α, VEGF, PDGF, and PI3K/PKB/mTOR (Phosphoinositide 3-kinases/Protein Kinase B) signaling (1, 4–7, 9). Several VEGF pathway inhibitors have been approved for the treatment of metastatic RCC, including sunitinib, bevacizumab, pazopanib, axitinib, and cabozantinib (22, 23, 26–30). As a result of alternative splicing of the eight-exon VEGF-A gene, VEGF-A presents several isoforms, and its expression is associated with both histology and prognosis (45). Multiple VEGF receptors have also been identified. While VEGFR1, VEGFR2 are expressed on vascular endothelial cells, VEGFR3 is expressed on lymphatic endothelial cells (46). VEGFR2 is the primary transducer of extracellular VEGF, mediating endothelial cell proliferation, migration, and resistance to apoptosis (47). Alternative splicing of *KDR*, the gene that encodes VEGFR2, results in a soluble 679-amino acid truncated extracellular-domain product (sVEGFR2) (48). Multiple clinical studies have shown associations between markers of VEGF activation and outcomes in patients treated with sorafenib and sunitinib. In a retrospective analysis of 903 RCC patients randomized to sorafenib vs. placebo, baseline VEGF levels were associated with both PFS and OS in univariate analysis (PFS, *P* = 0.0013; OS, *P* = 0.0009) (49). In a population of 63 patients receiving sunitinib, variations of serum levels of both sVEGFR2 (soluble VEGFR2) and sVEGFR3 during treatment correlated significantly with the objective response rate (ORR) (50). In another study conducted in patients receiving sunitinib after prior bevacizumab, low baseline levels of sVEGFR3 was also predictive of longer PFS (51).

Apart from VEGF-A, other soluble factors of predictive and prognostic value include multiple cytokines [e.g., IL-6, that can be directly secreted by cancer cells (52)] that have been variously implicated in the neoplastic process. In a study population involving 344 RCC patients randomized to either pazopanib or placebo in a phase III trial, serum concentrations at baseline of IL-8, hepatocyte growth factor (HGF), IL-6 and tissue inhibitor of metalloproteinases (TIMP)-1 were associated with a worse prognosis independently on the treatment arm, with some findings suggesting that baseline cytokine levels may be associated with a distinct sensitivity to pazopanib (53). In fact, patients with low vs. high baseline IL-6 levels showed a HR for survival favoring pazopanib compared to placebo of 0.55 vs. 0.31 (52). Importantly, IL-6, TIMP-1 and osteopontin were successfully incorporated in a prognostic model including five clinical variables and showing improved accuracy with respect to the Heng model, with a concordance-index of 0.75 vs. 0.67, respectively (54).

### Genetic Biomarkers

Several genetic factors have been investigated in RCC, but none of them have been assessed in randomized clinical trials (55, 56). Specific gene expression and single nucleotide polymorphisms (SNPs) can predict activity of TTs. Some studies suggest that SNPs in vascular endothelial growth factor receptor 3 (VEGFR3), cytochrome P450 3A5 (CYP3A5*1), IL-8, fibroblast growth factor receptor 2 (FGFR2), nuclear receptor subfamily 1 group I member 2 (NR1I2), and ATP binding cassette subfamily B member 1 (ABCB1) may predict efficacy and tolerance. No molecular/genetic biomarker has been validated in prospective clinical trials and can be used in clinical practice.

Some of the most studied genetic markers in RCC are:

- VHL
- SW1/SNF chromatin remodeling complex gene polybromo 1 (PBRM1)
- BRCA1 associated protein-1 (BAP1)

More than 90% of sporadic ccRCCs present loss of function of VHL, a tumor suppressor gene located on chromosome 3p. In normal tissue, VHL causes proteolysis of HIF-1α, but in RCC, VHL inactivation is associated with higher levels of HIF-1α, increased transcription of genes implicated in angiogenesis and tumorigenesis, such as VEGF and PDGF as well as activation of the PI3-K/PKB/mTOR pathway, that is involved in cancer progression (57). VHL loss is often the result of gene mutation or promoter hyper-methylation in RCC, but its implications in response to treatment are unknown. Choueiri et al. attempted to assess the relationship between VHL gene status and clinical outcomes to evaluate mRCC patients receiving VEGFR inhibitors. The response rate (RR) was 41% in mRCC patients with VHL inactivation vs. 31% in the wild-type VHL subgroup (*p* = 0.34). The presence of aberrant VHL gene, particularly loss-of-function mutations (frameshift, nonsense, splice, and in-frame deletions/insertions) corresponded to a 52% of RR. Thus, VHL loss-of-function mutations represent an independent prognostic factor linked to improved RR, but do not reflect progression-free survival (PFS) or OS. Similar findings were obtained for patients treated with both pazopanib and sorafenib (58–61). The PBRM1 /BAF180 tumor suppressor gene is mutated in 30–50% of ccRCC cases (62–64). PBRM1 is implicated in cell proliferation (65), and is found mutated at early stages of ccRCC (35). Mutations of PBRM1 are reported in small (<4 cm) RCC masses with an aggressive clinical behavior. Mutations of BAP1, a ubiquitin carboxyl-terminal hydrolase, correlate with negative histologic features (e.g., high nuclear grade) and poor cancer-specific survival. Inactivating mutations are reported in 15% of ccRCC. Mutation in the BAF180 gene excludes mutation of the BAP1 gene in most cases. Patients with BAP1-mutant vs. those with PBRM1-mutant RCC have shorter survival (4.6 years, 95% CI 2.1–7.2 vs. 10.6 years, 95% CI 9.8–11.5; HR = 2.7; 95% CI 0.99–7.6, *p* = 0.044) (26, 66). Furthermore, patients with BAP1-mutated tumors vs. PBRM1-mutated tumors are at higher risk of presenting metastatic dissemination (*p* < 0.023) and locally advanced disease (*p* < 0.042). In patients with localized RCC at diagnosis, a shorter relapse-free survival (RFS) was reported in BAP1 mutated disease (*p* = 0.059), with an uncertain association with survival (67, 68). BAP1 and PBRM1 appear as promising genetic prognostic markers for RCC and require prospective validation.

### Single-Nucleotide Polymorphisms

As recently shown, there are some single nucleotide polymorphisms (SNPs) (e.g., in genes involved in the VEGF pathway) that are associated with the risk of RCC (69, 70) as well as with RR and adverse events in patients receiving TTs. An analysis of 397 RCC patients receiving pazopanib assessed the clinical significance of 27 polymorphisms in 13 genes involved in angiogenesis, metabolism and drug transport. Three SNPs in IL-8 and HIF-1α and five SNPs in HIF-1α, NR1I2, and VEGFA were significantly associated with PFS and RR (*p* ≤ 0.05). RR was lower in patients with the VEGFA 1498CC vs. 1498TT genotype (33 vs. 51%, *p* ≤ 0.05) (71). Median PFS was significantly shorter in patients with two IL-8 polymorphisms associated with higher IL-8 gene expression than in those with wild-type IL-8 (27 vs. 48 weeks) (72). Furthermore, IL-8 has can potentially mediate resistance to TKIs (73). Variant alleles of IL-8 polymorphisms have been associated with worse OS in pazopanib- or sunitinib-treated mRCC patients (74). Another prospective study assessed response and toxicity to sunitinib in patients with ccRCC. Of the sixteen polymorphisms assessed in nine genes, Two VEGFR3 missense polymorphisms predicted a shorter PFS and a variant of CYP3A5*1 predicted higher toxicity at multivariate analysis. While VEGF or VEGFR SNPs were associated with outcomes, patients presenting VEGF SNP 936 and VEGFR2 SNP 889 had longer OS after adjusting for their risk group (*p* = 0.03) (75, 76). A retrospective study including 136 patients with metastatic ccRCC receiving sunitinib showed that SNPs in CYP3A5, ligand-activated nuclear receptor NR1I3, and ABCB1VEGF and VEGFR-2 were able to predict survival (77). Since most SNPs found to be associated with outcome have a low frequency, their impact in clinical practice is to be further ascertained, especially in-patient populations including different ethnicities. In an analysis of 138 VEGF SNPs assessed in patients treated with bevacizumab enrolled in the AVOREN trial (78) VEGFR1 SNP codifying for aminoacids located in its tyrosine-kinase domain associated with poor PFS (HR = 1.81, 95% CI 1.08–3.05, *p* = 0.033), although no association was found with OS (HR = 0.91, 95% CI 0.45–1.82, *p* = 0.78). In patients receiving axitinib in the AXIS (Comparative effectiveness of axitinib vs. sorafenib in advanced renal cell carcinoma) trial those with VEGFA rs699947 and rs833061 polymorphisms showed longer OS (27.0 vs. 13.4 months, HR = 0.39, *p* = 0.015), while those positive for the VEGFR2 rs2071559 polymorphism treated with sorafenib in this trial, had longer OS (26.8 vs. 13.8 months, HR = 0.41, *p* = 0.030). At multivariate analysis only VEGFR2 rs2071559 predicted PFS (*p* = 0.0053) and OS (*p* = 0.0027) in patients receiving sorafenib (45). Another study including 121 mRCC patients receiving sunitinib found the VEGFR1 SNP rs9582036 to be associated with OS (47). VEGFA SNPs have been associated with axitinib efficacy (78, 79), while SNPs in VEGFR3 have been associated with sunitinib efficacy (74). A recent meta-analysis by Liu et al. (80) suggested that, although some VEGFR1 genetic variants, such as VEGFR1 rs9582036 and rs9554320, were involved in sunitinib therapy outcomes, their clinical use as predictive biomarkers was limited, considering the negative results of all existing studies.

### MicroRNAs (miRNAs)

MicroRNAs are single-stranded RNAs that are not transduced into proteins, but affect gene expression at a post-transcriptional level and play a role in multiple pathologic processes, including cell growth (such as VHL), angiogenesis, apoptosis in RCC (81). High expression levels of a miRNA named miR-210 were associated with favorable pathologic features ccRCC patients in one study (82), while in others they correlated with increased risk of disease recurrence and shorter OS (83, 84). Lower miR-215 and miR-126 expression levels were associated with poor outcomes, whereas higher miR-126 expression levels were associated with a significantly longer disease-free survival and OS (85). One study found that simultaneous assessment of miR-21-5p, 142-3p, let-7g-5p, let- 7i-5p, and 424-5p and miR-204-5p could predict stage, grade, and time to progression ccRCC (86). Upregulation of miR942, miR-133a, miR-628-5p, and miR-484 was associated with sunitinib resistance in mRCC patients (87). Similarly, miR-141 downregulation was associated with epithelial-to-mesenchymal transition in ccRCC and poorer sunitinib response (88).

### Molecular Sub-classifications

ccRCC is an extremely complex and heterogeneous neoplasia involves different aberrant genes included VHL, TP53, chromatin modifier genes (PBRM1, SETD2, BAP1, ARID1A, MLL3, KDM5C, SMARCB1), PI3K/AKT/mTOR pathway genes (MTOR, PTEN, PIK3CA), MET, Hippo pathway gene NF2, NRF2-ARE pathway gene NFE2L2, and cell cycle genes (CDKN2A) (89). The understanding of the biomolecular processes involved in the proliferation and progression of ccRCC, allowed the identification of molecular subgroups with a prognostic and predictive value. First studies were conducted in non-metastatic ccRCC after nephrectomy in order to create a suitable prognostic model to predict the risk of recurrence. Brannon et al. identified two subtypes of RCC based on gene expression microarray data of 48 RCC: (1) ccA and (2) ccB with different biological signatures and prognoses. ccA tumors had a better prognosis than ccB, due to the overexpression of genes associated with hypoxia, angiogenesis, fatty acid metabolism, and organic acid metabolism, whereas ccB tumors overexpressed different aberrant genes that regulate EMT (epithelial–mesenchymal transition), identifying a more aggressive and immature subgroup (90). A subsequent meta-analysis of 480 ccRCC confirmed this classification, introducing a third cluster correlated with a wild type (WT) VHL expression profile and a non–clear cell phenotype (91). To standardize this model, Brooks et al. developed a novel molecular tool including 34-gene expression signature (ClearCode34) in 380 non-metastatic ccRCC patients from the TCGA dataset. At univariate analysis in the ccB subgroup a more recurrence rate occurred than ccA subgroup (HR: 2.3; 95% CI, 1.6–3.3; *p* = 4.3 × 10–6), with a higher risk of death from disease (HR, 2.9; 95% CI, 1.6–5.6; *p* = 0.0005) and from any cause vs. ccA subgroup (HR: 2.4; 95% CI, 1.6–3.7; *p* = 2.3 × 10–5) (92). The KIRC analysis working group evaluated mRNA/miRNA expression signatures on 446 ccRCC patients. An unsupervised clustering method identified four subsets (m1–m4), correlating with the prior reported ccA and ccB classification, particularly cluster m1 overlapped with the ccA group and, while ccB group corresponded to m2 and m3 sub-groups. Cluster m4 probably corresponded to the 15%, not included in the previous ccA/ccB classification (93). Subsequently, Chen et al. refined this classification, identifying three different subtypes of predominantly ccRCC carcinoma, CC-e.1, CC-e.2, CC-e.3, correlating with intermediate, better and worse OS, respectively. The previously reported ccA and ccB classification correlated with CC-e.2 (better prognosis) and CC-e.3 (worse prognosis), respectively; instead, considering the KIRC subtypes, m1 and m3 corresponded to CC-e.2 and CC-e.3, respectively, while CC-e.1 correlated with m2 and m4 (89).

Combining the results of these studies, we identify three main significant prognostic groups:

1. Good prognosis: ccA, CC-e.2, and m1 groups, involved gene sets associated with chromatin remodeling processes and a higher frequency of PBRM1 mutations;
2. Poor prognosis: ccB, CC-e.3, m3 groups, characterized by higher expression of cell cycle genes (CDKN2A) and hypoxia-related genes, EMT, hypermethylation, chromatin modifier genes mutation (SETD2 or BAP1), PI3K/AKT/mTOR pathway genes mutations (PTEN), and a metabolic shift (high glutathione and high dipeptide levels) (94);
3. Intermediate prognosis: cluster 3, CC-e.1, m2, and m4, characterized by higher frequencies of BAP1 mutations and base-excision repair.

This prognostic molecular sub-classification was validated in non-metastatic patients, so how can these molecular sub-classifications predict the response to VEGFRi in mccRCC? de Velasco et al. evaluated the predictive value of ClearCode34 in the setting of systemic mccRCC treatment, showing a longer mOS for low-risk-ccA than high-risk-ccB subtypes [27.6 vs.22.3 months (HR: 2.33; *p* = 0.039), respectively]. On multivariable analyses and adjusting for IMDC groups, ccB remained associated with a worse OS (*p* = 0.044) (95). Recently, Beuselinck et al. identified four molecular ccRCC subgroups correlating with VEGFRi (sunitinib) treatment: (1) ccrcc1 (“c-myc-up”) and ccrcc4 (“c-myc-up and immune-up”) with shorter PFS, OS and poorer response to sunitinib, (2) ccrcc2 (“classical”) and ccrcc3 (“normal-like”) with longer OS and better sunitinib response (*p* < 0.0001). Moreover, these four ccrcc groups correlated with the three subtypes ccA, ccB, and cluster-3 described by Brannon et al., particularly the poor-prognosis ccB group included ccrcc1 and ccrcc4 subgroups. The ccrcc1/ccrcc4 subtypes, resulted non-responders to sunitinib, and expressed common molecular characteristics like upregulation of MYC targets or a hypermethylated status associated with a less differentiated (76% of Fuhrman grade 4) phenotype. PBRM1 and aberrant VHL gene were most frequently identified in ccrcc1/ccrcc2. The ccrcc4 had higher inflammation score, sarcomatoid dedifferentiation, mutated BAP1, low frequency of aberrant VHL and wilde type PBRM1 (96). Patients labeled with ccrcc2-group had longer mPFS (20 months) and mOS (35 months) comparable to mPFS (24 months) and mOS (40 months) of ccrcc3-group, while the ccrcc1-group reached intermediate outcome (mPFS 12 months and mOS 22 months)and, the ccrcc4-group the poorest outcome (mPFS 8 months and mOS 14 months). To explain these different outcomes, an mRNA-expression of genes associated with angiogenesis was analyzed. The ccrcc2-3-group displayed the highest expression of the pro-angiogenic HIF-VEGF-VEGFR-pathway (HIF2A, VEGFA, VEGFR1, VEGFR2, and VEGFR3), particularly in tumors with a bi-allelic PBRM1 inactivation. In the poor responding ccrcc4-group, the neo-angiogenesis was poorly expressed (97). These data were confirmed by Verbiest et al., who showed a mPFS of 9 months for the ccrcc2 and ccrcc3, 5 months for ccrcc1, and 3 months for the ccrcc4-group, respectively (*P* = 0.011), with a mOS of 69, 19, and 5 months, respectively (*P* = 0.003). The ccrcc1-4 classification becomes a predictor of outcomes with VEGFRi (sunitinib and pazopanib) in the metastatic setting (98, 99). Angiogenesis expression signatures correlate with outcomes on VEGFRi treatments: high Angioscore correlates with good outcomes, generally associated with loss of PBRM1, conversely BAP1 loss associates with decreased angiogenic signaling and poor outcomes (100). Is there an overlap between these molecular sub-classifications and the IMDC risk groups, commonly used in clinical practice? Verbiest et al. correlated this molecular classification integrated with IMDC risk groups and sarcomatoid dedifferentiation, with the outcomes on first-line VEGFRi (sunitinib or pazopanib). The ccrcc2 group correlated with higher angiogenic gene expression, resulting more represented in IMDC good risk and poorly in IMDC poor risk (*p* < 0.001). The ccrcc2 group and angiogenic gene expression correlated positively with longer PFS in IMDC intermediate-risk patients too (*p* = 0.006; *p* = 0.04). The ccrcc4- subtype was typically grouped in IMDC poor risk group, with low angiogenic gene expression, often with sarcomatoid differentiation and poorest outcomes (101). Therefore, the Angio phenotype correlates with superior outcomes, regardless IMDC risk category, although enrichment in angiogenesis gene expression is more represented in the good IMDC and part of intermediate IMDC risk group. In the era of immunotherapy with the approval of immuno-checkpoint inhibitors, the molecular classification gets a decisive role both as a carrier for understanding the connection mechanisms between the various aberrant molecular pathways involved with the immune system and as a predictor of outcomes. The expression of the immune checkpoint molecule programmed death-ligand 1 (PD-L1) on tumor cells and/or tumor-infiltrating immune cells (IC) has been reported to inhibit antitumor immunity and correlated with poor prognosis in mccRCC. VEGF plays a central role in cancer immune evasion, therefore anti-VEGF might reinforce the antitumor activity of antiPD-L1 through the T-cell enhancement, upregulating the major histocompatibility complex class I expression, and reducing myeloid immunosuppression. The expression of regulatory cytokines (IL10, TGFB1) and T-cell immunosuppressive molecules [PD-L1-2, PD-1, LAG 3], the presence of myeloid-derived suppressor cells as well as regulatory T cells correlate with VEGFRi treatment failure (102–106). The Immune signature, like the Angioscore correlated with molecular subtype (*p* = 0.0007). Recently the Checkmate 214 showed the efficacy of Ipilimumab plus Nivolumab in mccRCC, across all risk groups, although VEGFRi (sunitinib) performed better in good IMDC risk groups, due to the higher angiogenesis expression signatures, typical of this group (107). The previous sub-classifications identified the ccrcc4 (Beuselinck et al.), like Cluster 4 established by Hakimi et al. in the COMPARZ trial (108), as an inflamed subtype with moderate angiogenesis, but high immune infiltration, PD-L1 expression on tumor cells by IHC, and poor response to VEGFRi. IMDC poor risk groups were enriched either in immune-exhausted ccrcc4 tumors or in immune-cold ccrcc1 tumors (101). Considering the strongly correlation between the angiogenesis and the immune-system activation, several phase III trials have explored the new treatment paradigm: the association between VEGF/VEGFRi and immuno-checkpoint inhibitors (107, 109–111), with superb results in all IMDC risk-groups, molecular sub-groups, particularly in VEGFRi refractory patients, IMDC poor risk groups. A biological subgroups analysis of the phase 2 IMmotion150 study correlated the expression levels of angiogenesis (Angio), immune (Teff) and myeloid inflammation-associated genes (Myeloid) with outcomes. VEGFRi (Sunitinib) efficacy was higher in angiogenic tumors (AngioHigh), while the combination of atezolizumab + bevacizumab showed a larger clinical benefit in TeffHigh and in TeffHighMyeloidLow, particularly in TeffHighMyeloidHigh in which atezolizumab monotherapy failed, supporting the role of VEGFRi to overcome innate inflammation-mediated resistance (112). This data was confirmed by the phase III IMmotion151 trial. High Teff gene expression signatures (GE) or low angiogenesis GE were associated with longer PFS for atezolizumab plus bevacizumab vs. sunitinib, HR 0.76; 95% CI, 0.59–0.99; conversely, the efficacy was the same in high angiogenesis GE (HR, 0.95; 95% CI, 0.75–1.19). An interesting point was the further confirmation that angiogenesis GE was higher in favorable vs. intermediate to poor MSKCC risk groups (*p* = 4.28e−06). The main limit of these molecular classifications and its use in clinical practice, was the significant intratumor and inter-tumor (metastases) heterogeneity (110). Several analyses suggest that different interactions occur between the primary tumor and metastatic sites, and the coexistence of various sub-clones with different prognosis, particularly in intermediate IMDC risk group. Therefore, identification of a single driver gene based on single regional sequencing significantly under-estimates the true molecular tumor-assessment. The evidence suggest that multiple biopsy samples included metastases site, occur for a complete molecular analysis and to identify the clone with poor prognostic features (113–115).

## Biomarkers Predictive of Adverse Events (AEs) of Anti-Angiogenic Therapy

Most adverse events associated with anti-angiogenic therapy are the result of known either on-target or off-target inhibitory effects on tyrosine kinases inhibitors (116). For this reason, AEs may be associated with outcomes in some cases (117).

### Hypertension

Hypertension is a commonly reported adverse event in mccRCC patients treated with anti-angiogenic therapy, and correlates with a systemic dysfunction of microcirculation, activation of the endothelin-1 system, suppression of the renin-angiotensin system, inhibition of endothelial nitric oxide synthase, and increased vascular stiffness (52, 53, 116). Hypertension was initially reported to be associated with bevacizumab (117). Notably, in a phase III trial of bevacizumab plus IFN-α vs. IFN-α alone in patients with mRCC, patients in the bevacizumab arm who developed grade 2 hypertension showed a significantly longer PFS (13.2 months, 95% CI 10.6–15.5 vs. 8.0 months, 95% CI 5.9–8.6, *p* = 0.001) and OS (41.6 months, 95% CI 26.3–55.1 vs. 16.2 months, 95% CI 14.2–18.7, *p* = 0.001) than those who did not (23). In the multiple studies that explored the relationship between anti-angiogenic therapy and hypertension, a varying incidence of all-grade and grade 3/4 hypertension was reported, possibly as a result of differences in trial populations and timing and protocols for measuring blood pressure (28–31). A retrospective review involving 3 clinical trials showed that hypertension was associated with longer PFS, OS, and ORR in patients treated with sunitinib (49, 50, 118, 119). Patients enrolled in the AXIS trial who reported a diastolic blood pressure ≥90 mmHg within the first 8 or 12 weeks of randomization had a longer survival independently on the treatment arm: 20.7 months (95% CI 18.4–24.6) vs. 12.9 months (95% CI 10.1–20.4) in the axitinib group (*p* = 0.01), and 20.2 months (95% CI 17.1–32.0) vs. 14.8 months (95% CI 12.0–17.7) in the sorafenib group (*p* = 0.002) (77). These findings could not be confirmed by the Italian SAX study on real-world use of axitinib (120). A recent retrospective study showed that grade 3 hypertension affected positively OS in patients treated with pazopanib in real-world settings (HR = 0.22, 95% CI 0.05–0.8, *p* = 0.03) (121).

### Hypothyroidism

Hypothyroidism is another frequent but generally mild adverse event known to be caused by anti-VEGFR TKIs (122, 123). The underlying biology of this adverse event includes multiple events/factors such as destructive thyroiditis, endothelial dysfunction, impaired iodine uptake, and reduced synthesis of thyroid hormone (122–127). Hypothyroidism has also been associated with outcome in patients receiving anti-angiogenic therapy. In one retrospective study by Wolter et al. (128) conducted in 40 mRCC patients, a longer mPFS (10.3 vs. 3.6 months) and mOS (18.2 vs. 6.6 months) were reported in patients with thyroid dysfunction than in those with normal thyroid function. Subclinical hypothyroidism diagnosed during the first 2 months of treatment has also been reported to be associated with survival (128–130). Inconsistent results were reported in a prospective study of 111 mRCC patients treated with sunitinib, that did not find any relationship between abnormal thyroid function and PFS (18.9 vs. 15.9 months) (131). Furthermore, one meta-analysis including 11 retrospective and prospective studies enrolling mRCC patients receiving sunitinib or sorafenib failed to identify any predictive value of hypothyroidism (HR for progression = 0.82, 95% CI 0.59–1.13, *p* = 0.22; 6 studies, 250 patients) (132). Thyroid dysfunction (both hypo and hyperthyroidism) may be associated with a longer PFS (HR = 0.12, 95% CI 0.02–0.78, *p* = 0.02) in patients treated with pazopanib (121).

### Hand-Foot Syndrome

Hand-foot syndrome of any grade is approximately reported in up to half of patients receiving TKIs, with 9% patients showing grade 3 or 4 hand and foot syndrome. This adverse event is related to dermal endothelial cell apoptosis due to inhibition of VEGFR and PDGFR (platelet-derived growth factor receptor) in (133–135) TKIs may also mediate skin toxicity when secreted in the eccrine glands of the skin, which are rich in c-KIT (128–130). Pone retrospective study reported hand-foot syndrome to be associated with improved OS, PFS, and ORR. Hand-foot syndrome is able to predict PFS and OS (136).

### Fatigue

Fatigue is a commonly reported adverse event in patients treated with TKIs. Fatigue may be caused by the systemic inflammatory state related to the underlying malignancy, concomitant use of medications, anemia, or hypothyroidism and hypopituitarism (137). It was reported in up to half of treated patients (138). Patients with fatigue or asthenia seem to have improved clinical outcomes in terms of PFS and OS, although this may be the result of a longer on trial time (139, 140).

## Optimization of Treatment Sequence

At present time, there are no definitive data supporting one specific treatment sequence over the others, with multiple drugs recommended in the first, second and third-line setting (Table 3). In the new era of Immunotherapy, are VEGF-TKIs still a valid option for mccRCC treatment? The angiogenesis plays a central role in the ccRCC tumorigenesis and progression, regulating the immune landscape through abnormal tumor vessel formation, dysregulation of various immune cells and promoting an immunosuppressive tumor microenvironment. Therefore, anti-angiogenic therapy (sunitinib, pazopanib) remains a valid option in selected patients (VEGF-dependent favorable IMDC risk group first-line mccRCC) and enhances the activity of immunotherapy, modulating immune response. Different phase 3 trials evaluated or are evaluating combination of immune checkpoint inhibitors, such as anti PD-1 nivolumab and anti CTLA-4 ipilimumab, or anti PD-1/PDL-1 and VEGF/VEGFRi in first-line treatment, with superb results that will consequently change the therapeutic sequence in the first and second-line. The combination of VEGF/VEGFRi plus immune checkpoint inhibitors (IOVE) or Ipilimumab-Nivolumab (IO) will represent the gold standard treatment in first-line setting, across all IMDC risk group, particularly in poor risk patients with low angiogenic gene expression and sarcomatoid dedifferentiation. No data exist on the best first-line therapy. Recently Dudani et al., using the IMDC dataset, showed no significant differences in first-line outcomes for patients receiving IOVE combination vs. IO combination. (141). Hahn et al. conducted a Systematic Review and Network Meta-analysis of First-line Treatment of mccRCC. In the ITT population, cabozantinib [surface under the cumulative ranking curves [SUCRA] 84%], avelumab plus axitinib (SUCRA 68%), and pembrolizumab plus axitinib (SUCRA 82%) were superior to the other agents for PFS; pembrolizumab plus axitinib appeared superior for OS (SUCRA 95%); and atezolizumab demonstrated the lowest likelihood of AEs (SUCRA 100%). Findings were similar in the intermediate/poor-risk subgroup. Avelumab plus axitinib may be preferred in patients with favorable-risk disease. It is less clear what the superior treatment is for ORR, but avelumab plus axitinib, cabozantinib, and pembrolizumab plus axitinib performed better than other comparators (142). Until the recent past, clinicians selected second- and third-line therapies based on response to first-line therapy. Nevertheless, a retrospective review conducted by the IMDC demonstrated no correlation between both first- and second-line PFS (Pearson correlation coefficient 0.025, *p* = 0.59)and first- and second-line ORR (chi-squared trend test *p* = 0.17) (143). Elaidi et al. (144) showed that patients who remained on first-line TKI during 11–22 months benefited from a TKI re-challenge, rather than from second-line mTOR inhibitors (HR ≈ 0.5), with a median PFS of 9.4 months (5.9–12.2) vs. 3.9 months (3.0–5.5) (*p* = 0.003), whereas time-to-treatment failure was 8.0 months (5.5–11.0) vs. 3.6 months (3.0–4.6) (*p* = 0.009). PFS improved more with second-line VEGFR TKI if first-line VEFGR TKI was given for >8 months (11.3 vs. 5.1 months, *p* = 0.009), suggesting continued VEGF inhibition may be a pertinent strategy in this subset of patients. The duration of first-line PFS is an independent prognostic variable but it is not predictive for PFS associated with subsequent therapy (145). Other authors found that the response and the PFS to a first-line TKI correlated with longer PFS and OS using everolimus as second-line treatment (146). The tumor shrinkage remains a prognostic factor, regardless of first-line therapy, and maximal tumor shrinkage (60–100%) was an independent predictor of longer OS (147). These data were confirmed by Basappa et al. (148), who identified a total tumor burden <13 cm (*p* = 0.09) as an independent positive predictor of PFS, and a baseline number of metastases <10 (*p* < 0.001) and tumor burden above the diaphragm <6.5 cm (*p* = 0.05) as independent predictors of OS. Tumor shrinkage associated significantly with longer OS (*p* < 0.001) in patients receiving sunitinib. The lack of response to a VEGF/VEGFRi not preclude positive clinical outcomes on second-line VEGF/VEGFRi. A *post-hoc* analysis of the AXIS trial did not show significant differences in PFS or OS in responders vs. non-responders, as well as according to the duration of first-line sunitinib treatment; conversely the mOS was longer in patients with smaller vs. larger tumor burden, except in those treated with the cytokine-axitinib sequence (149). Furthermore, in the AXIS trial, the following factors were found to be associated with shorter OS: prior treatment with sunitinib, ECOG performance status ≥ 1, <1 year from initial diagnosis to the first treatment, more than one metastatic site, particularly liver or bone metastases, anemia, neutrophilia, hypercalcemia or high level of LDH and alkaline phosphatase. These findings were confirmed by the RECORD-1 trial (150). Two additional studies concluded that everolimus treatment prolonged PFS, if the patient had received only one rather than two prior TKIs (151). Overall, the chosen systemic regimens, PFS, intensity and duration of response do not influence selection of the optimal sequence. The greater benefit to mRCC patients comes from an adequate sequential administration of available systemic regimens. Poor risk patients or non-responders show worse OS, PFS, and ORR due to the biology/aggressiveness of the tumor rather than sensitivity of the disease to a specific agent. Analysis of phase II and III trials of nivolumab in pretreated mRCC patients, showed longer OS regardless of first-line treatment (sunitinib, pazopanib, and IL-2), duration of first-line therapy (<6 or ≥6 months). Conversely, the number of prior systemic regimens affected the OS: a longer mOS (not reached, 95% CI 19.8–not reached) was associated with only one previous treatment vs. two or more prior regimens (18.7 months, 95% CI 13.4–26, p not shown) (104, 152). Recently the results of two other trials evaluating, cabozantinib, and lenvatinib plus everolimus, showed superior RR and longer OS in second-line setting (27, 104, 153, 154). Treatment selection in this setting, is based on different factors, such as patient PS, contraindications and comorbidities, RCC subtype, safety profiles, and previous treatments. Bracarda et al. published a prognostic factor analyses from the AXIS trial, identifying axitinib as effective (post sunitinib) 2nd line therapy option for mccRCC with VEGF-dependent mRCC (AngioHigh), good/intermediate risk group, low tumor burden, without bone or liver metastases (155). In the new era of first line Immunotherapy, are VEGF-TKIs still a valid option for second-line mRCC treatment? To date no data, exist about post Immunotherapy (IO-IOVE) first-line therapy. Recently Auvray et al. reported the clinical outcomes of second line TKI, as part of Checkmate 214, showing 12 PR (36%), 13 SD (39%) and 5 PD (15%), evaluating 30 patients received subsequent TKI. The mPFS was 8 months (5–13), particularly 8 months (5–16) with sunitinib or pazopanib and 7 months (5–NA) with axitinib or cabozantinib. Overall PFS resulted longer in patients with long first-line response duration (≥6 months) vs. short response duration (<6 months): 8 vs. 5 months (*p* = 0.03), respectively. Interesting, OS rate was 54% at 12 months. Toxicity was as expected: 42% developed at least one toxicity grade ≥3 (156). A retrospective study of mccRCC patients treated with second-line (2L) VEGFRi after first-line ICI. 2L TKI therapies included pazopanib, sunitinib, axitinib and cabozantinib. On 2L TKI therapy, one patient (1.5%) achieved a CR, 27 patients (39.7%) a PR and 36 patients (52.9%) SD. Median PFS was 13.2 months (95% CI: 10.1, NA). Forty-five percent of subjects required a dose reduction, and 27% of patients discontinued treatment because of toxicity (157). The efficacy and safety of VEGFR-TKIs after PD-1/PD-L1 inhibition were demonstrated by Nadal et al. in a retrospective study; as expected, the RR was lower and the mPFS was shorter in those patients who received prior anti PD-1/PD-L1 plus VEGF/VEGFRi vs. patients treated with prior IO alone (158). Prospective trial will be needed to re-assess the new sequence of anti PD-1/PD-L1 and VEGF/VEGFRi and their combination, particularly in the second-line setting, mostly after the impressive results of the combination trials of immune checkpoint inhibitors and immune checkpoint inhibitors with VEGF/VEGFRi in the first-line therapy.

**Table 3:** Medical treatment of mRCC according to EAU guidelines.

**IMDC risk group**	**First-line therapy**	**Second-line therapy**	**Third line therapy**
Favorable	Sunitinib or pazopanib	Cabozantinib or nivolumab	Cabozantinib or nivolumab
Intermediate and poor	Ipilimumab+nivolumab Cabozantinib, sunitinib or pazopanib	Cabozantinib or other VEGF targeted therapy VEGF targeted therapy or nivolumab	Cabozantinib or other targeted therapy Other targeted therapy or nivolumab

*European Association of Urology guidelines accessed at https://uroweb.org/guideline/renal-cell-carcinoma/#7 on 6th May 2019. VEGF, vascular endothelial growth factor; IMDC, International Metastatic RCC Database Consortium*.

## Conclusions

As summarized in Table 4, we reviewed the most promising predictive and prognostic available biomarkers in RCC patients treated with antiangiogenic agents. With the advent of novel immunotherapy agents, the clinical need to personalize treatment has become more compelling. At the present time, there are no effective biomarkers that can be incorporated in the therapeutic algorithm, despite large research efforts. Most available data come from large retrospective analyses or even small samples and can only establish a trend, but require prospective validation in order to be considered practice-changing. Novel genomic and transcriptomic biomarkers, such as circulating tumor DNA and miRNA in serum or plasma, have great potential to become practice-changing in the future, as they can reflect deep aspects of tumor biology and can be assessed non-invasively and at decreasing costs.

**Table 4:** Main prognostic and predictive statistically significant biomarkers reviewed.

**References**	**Patient population**	**Biomarker(s)**	**Sample size**	**Main findings**
D' Alterio et al. (41)	mRCC	CXCR4 Negative/Low vs. Mod vs. High expression	62	HR for progression in patients treated with sunitinib = 2.04; *p* = 0.0271
				HR for death in patients treated with sunitinib = 1.48 (95%CI = 0.93–2.38); *p* = 0.0974
Motzer et al. (43)	advanced RCC	HIF1a low vs. high expression	292	HR for progression in patients treated with sunitinib = 1.55; *p* = 0.0341
				HR for death in patients treated with sunitinib = 1.34; *p* = 0.2095
Rini et al. (51)	mRCC	sVEGFR-3 less than median baseline value vs. greater than median serum levels	59	HR for progression in patients treated with sunitinib = 0.45; *p* = 0.006
		VEGF-C less than median baseline value vs. greater than median serum levels	57	HR for progression in patients treated wit sunitinib = 0.37; *p* = 0.0006
Tran et al. (53)	mRCC	Interleukin 6 low serum levels	344	HR for progression in patients treated with pazopanib vs. interferon = 0.55; *p* = 0.009
		Interleukin 6 high serum levels		HR for progression in patients treated with pazopanib vs. interferon = 0.31; *p* = 0.009
		Interleukin 6 low serum levels		HR for death in patients treated with pazopanib vs. interferon = 1.41; *p* = 0.005
		Interleukin 6 high serum levels		HR for death in patients treated with pazopanib vs. interferon = 0.42; *p* = 0.005
		Interleukin 8 low serum levels		HR for death in patients treated with pazopanib vs. interferon = 1.49; *p* = 0.002
		Interleukin 8 high serum levels		HR for death in patients treated with pazopanib vs. interferon = 0.42; *p* = 0.002
		Osteopontin low serum levels		HR for death in patients treated with pazopanib vs. interferon = 0.96; *p* = 0.033
		Osteopontin high serum levels		HR for death in patients treated with pazopanib vs. interferon = 0.41; *p* = 0.033
		VEGF low serum levels		HR for death in patients treated with pazopanib vs. interferon = 1.2; *p* = 0.006
		VEGF high serum levels		HR for death in patients treated with pazopanib vs. interferon = 0.41; *p* = 0.006
Peña et al. (62)	Advanced RCC	VEGF high vs. low expression	348	HR for death in patients treated with sorafenib = 1.64; *p* = 0.0027
		CAIX high vs. low expression	66	HR for death in patients treated with sorafenib = 2.26; *p* = 0.034
		TIMP-1 high vs. low expression	63	HR for death in patients treated with sorafenib = 3.34; *p* = 0.001
		Ras p21 high vs. low expression	65	HR for death in patients treated with sorafenib = 2.49; *p* = 0.016
Xu et al. (72)	Advanced/mRCC	IL8 2767TT genotype vs. IL8 wild-type AA genotype	397	HR for progression in patients treated with pazopanib = 1.8; *p* = 0.009
		IL8 251AA genotype vs. IL8 wild-type TT genotype		HR for progression in patients treated with pazopanib = 1.7; *p* = 0.01
		HIF1a 1790AG genotype vs. HIF1a wild type GG genotype		HR for progression in patients treated with pazopanib = 1.8; *p* = 0.03
Xu et al. (74)	Advanced RCC	IL8 rs1126647 polymorphism, variant T allele vs. reference variant A	186	HR for death in patients treated with pazopanib = 1.45; *p* = 0.007
			337	HR for death in patients treated with sunitinib = 1.39; *p* = 0.008
			88	HR for death in patients treated with sunitinib = 1.62; *p* = 0.034
Garcia-Donas et al. (75)	Advanced RCC	VEGFR3 rs307826 AA variant vs. AG varian	95	HR for progression in patients treated with sunitinib = 3.57; *p* = 0.0079
		VEGFR3 rs307821 GG variant vs. GT variant		HR for progression in patients treated with sunitinib = 3.31 (95% CI = 1.64–6.68); *p* = 0.014
van der Veldt et al. (77)	mRCC	ABCB1 other haplotypes vs. TCG haplotype	129	HR for progression in patients treated with sunitinib = 0.52; *p* = 0.033
		CYP3A5 6986A/G GG haplotype vs. AG+AA haplotypes	128	HR for progression in patients treated with sunitinib = 0.26; *p* = 0.032
Escudier et al. (78)	mRCC	VEGFR2 rs2071559 AA variant vs. GG variant	141	HR for progression in patients treated with sorafenib = 2.22; *p* = 0.0053
		VEGFR2 rs2071559 AA variant vs. GG variant		HR for death in patients treated with sorafenib = 2.58; *p* = 0.0027
		VEGFR2 rs1870377 TT variant vs. TA variant	47	HR for progression in patients treated with sorafenib = 0.62; *p* = 0.026
		HIF-1α rs11549465 CC variant vs. CT variant	33	HR for progression in patients treated with axitinib = 1.93; *p* = 0.006
				HR for death in patients treated with axitinib = 1.88; *p* = 0.007
		VEGF-A rs699947 CC variant vs. AA variant	42	HR for death in patients treated with axitinib = 0.39; *p* = 0.001

*mRCC, metastatic renal cell carcinoma; VEGF, vascular endothelial growth factor; CXCR, chemokine receptor; HIF, hypoxia-inducible factors; VEGFR, vascular endothelial growth factor receptor; TIMP−1, metallopeptidase inhibitor 1; KDR, kinase insert domain receptor; CAIX, Carbonic Anhydrase IX; IL, interleukin; ABCB1, ATP binding cassette subfamily B member 1; CYP3A5, cytochrome P450 family 3 subfamily A member; ABCG2, ATP-binding cassette super-family G member*.

## Author Contributions

All authors listed have made a substantial, direct and intellectual contribution to the work, and approved it for publication.

### Conflict of Interest

The authors declare that the research was conducted in the absence of any commercial or financial relationships that could be construed as a potential conflict of interest.

## References

[1] Escudier B Albiges L Sonpavde G. Optimal management of metastatic renal cell carcinoma: current status. Drugs. (2013) 73:427–38. 10.1007/s40265-013-0043-1 23572408

[2] Rodriguez-Vida A Strijbos M Hutson T. Predictive and prognostic biomarkers of targeted agents and modern immunotherapy in renal cell carcinoma. ESMO Open. (2016) 1:e000013. 10.1136/esmoopen-2015-000013 27843601 PMC5070260

[3] Cohen HT McGovern FJ. Renal-cell carcinoma. N Engl J Med. (2005) 353:2477–90. 10.1056/NEJMra043172 16339096

[4] Dorević G Matusan-Ilijas K Babarović E Hadzisejdić I Grahovac M Grahovac B Hypoxia inducible factor-1-alpha correlates with vascular endothelial growth factor A and C indicating worse prognosis in clear cell renal cell carcinoma. J Exp Clin Cancer Res. (2009) 28:40 10.1186/1756-9966-28-40 19302703 PMC2664792

[5] Nicol D Hii SI Walsh M The B Thompson L Kennett C. Vascular endothelial growth factor expression is increased in renal cell carcinoma. J Urol. (1997) 157:1482–6. 10.1097/00005392-199704000-00108 9120987

[6] Kornakiewicz A Solarek W Bielecka ZF Lian F Szczylik C Czarnecka AM. Mammalian target of rapamycin inhibitors resistance mechanisms in clear cell renal cell carcinoma. Curr Signal Transduct Ther. (2014) 8:210–8. 10.2174/1574362409666140206222746 25152703 PMC4141323

[7] Dimova I Popivanov G Djonov V. Angiogenesis in cancer - general pathways and their therapeutic implications. J BUON. (2014) 19:15–21. 24659637

[8] McDermott DF Rini BI. Immunotherapy for metastatic renal cell carcinoma. BJU Int. (2007) 99(5 Pt B):1282–8. 10.1111/j.1464-410X.2007.06818.x 17441925

[9] Mickley A Kovaleva O Kzhyshkowska J Gratchev A. Molecular and immunologic markers of kidney cancer-potential applications in predictive, preventive and personalized medicine. EPMA J. (2015) 6:20. 10.1186/s13167-015-0042-2 26500709 PMC4617448

[10] Buti S Bersanelli M Maines F Facchini G Gelsomino F Zustovich F First-line PAzopanib in non-clear-cell renal cArcinoMA: the italian retrospective multicenter PANORAMA study. Clin Genitourin Cancer. (2016) 27:832P 10.1093/annonc/mdw373.59 28108284

[11] Cecere SC Rossetti S Cavaliere C Della Pepa C Di Napoli M Crispo A. Corrigendum: pazopanib in metastatic renal cancer: a “real-world” experience at national cancer institute “Fondazione G. Pascale”. Front Pharmacol. (2016) 7:468. 10.3389/fphar.2016.00468 27924140 PMC5136557

[12] Di Lorenzo G De Placido S Pagliuca M Ferro M Lucarelli G Rossetti S. The evolving role of monoclonal antibodies in the treatment of patients with advanced renal cell carcinoma: a systematic review. Expert Opin Biol Ther. (2016) 16:1387–401. 10.1080/14712598.2016.1216964 27463642

[13] Di Lorenzo G Buonerba C Federico P Rescigno P Milella M Ortega C. Third-line sorafenib after sequential therapy with sunitinib and mTOR inhibitors in metastatic renal cell carcinoma. Eur Urol. (2010) 58:906–11. 10.1016/j.eururo.2010.09.008 20884115

[14] Frank I Blute ML Cheville JC Lohse CMM Weaver AL Zincke H. An outcome prediction model for patients with clear cell renal cell carcinoma treated with radical nephrectomy based on tumor stage, size, grade and necrosis: the SSIGN score. J Urol. (2002) 168:2395–400. 10.1016/S0022-5347(05)64153-5 12441925

[15] Negrier S Escudier B Gomez F Douillard JY Ravaud A Chevreau C. Prognostic factors of survival and rapid progression in 782 patients with metastatic renal carcinomas treated by cytokines: a report from the Groupe Francais d'Immunotherapie. Ann Oncol. (2002) 13:1460–68. 10.1093/annonc/mdf257 12196373

[16] Manola J Royston P Elson P McCormack JB Mazumdar M Négrier S. International Kidney Cancer Working Group. Prognostic model for survival in patients with metastatic renal cell carcinoma: results from the international kidney cancer working group. Clin Cancer Res. (2011) 17:5443–50. 10.1158/1078-0432.CCR-11-0553 21828239 PMC3387987

[17] Motzer RJ Mazumdar M Bacik J Russo P Berg WJ Metz EM. Effect of cytokine therapy on survival for patients with advanced renal cell carcinoma. J Clin Oncol. (2000) 18:1928–35. 10.1200/JCO.2000.18.9.1928 10784634

[18] Motzer RJ Bacik J Murphy BA Russo P Mazumdar M. Interferon-alfa as a comparative treatment for clinical trials of new therapies against advanced renal cell carcinoma. J Clin Oncol. (2002) 20:289–96. 10.1200/JCO.2002.20.1.289 11773181

[19] Motzer RJ Bacik J Schwartz LH Reuter V Russo P Marion S. Prognostic factors for survival in previously treated patients with metastatic renal cell carcinoma. J Clin Oncol. (2004) 22:454–63. 10.1200/JCO.2004.06.132 14752067

[20] Heng DY Xie W Regan MM Warren MA Golshayan AR Sahi C. Prognostic factors for overall survival in patients with metastatic renal cell carcinoma treated with vascular endothelial growth factor-targeted agents: results from a large, multicenter study. J Clin Oncol. (2009) 27:5794–99. 10.1200/JCO.2008.21.4809 19826129

[21] Heng DY Xie W Bjarnason GA A unified prognostic model for first- and second-line targeted therapy in metastatic renal cell carcinoma (mRCC): results from a large international study. Proc Am Soc Clin Oncol. (2010) 28(Suppl.):4523 10.1200/jco.2010.28.15_suppl.4523

[22] Rini BI Halabi S Rosenberg JE Stadler WM Vaena DA Ou SS. Bevacizumab plus interferon alfa compared with interferon alfa monotherapy in patients with metastatic renal cell carcinoma: CALGB 90206. J Clin Oncol. (2008) 26:5422–8. 10.1200/JCO.2008.16.9847 18936475 PMC2651074

[23] Escudier B Bellmunt J Negrier S Bajetta E Melichar B Bracarda S. Phase III trial of bevacizumab plus interferon alfa-2a in patients with metastatic renal cell carcinoma (AVOREN): final analysis of overall survival. J Clin Oncol. (2010) 28:2144–50. 10.1200/JCO.2009.26.7849 20368553

[24] Facchini G Perri F Caraglia M Pisano C Striano S Marra L. New treatment approaches in renal cell carcinoma. Anti-Cancer Drugs. (2009) 20:893–900. 10.1097/CAD.0b013e32833123d4 19752718

[25] D'Aniello C Cavaliere C Pisconti S Facchini G. Long-term response to pazopanib in an elderly man with mRCC. A case report. Tumori. (2014) 100:e305–8. 10.1177/1778.19308 25688517

[26] Motzer RJ Hutson TE Tomczak P Michaelson MD Bukowski RM Rixe O. Sunitinib versus interferon alfa in metastatic renal-cell carcinoma. N Engl J Med. (2007) 356:115–24. 10.1056/NEJMoa065044 17215529

[27] Choueiri TK Escudier B Powles T Tannir NM Mainwaring PN Rini BI METEOR investigators. Cabozantinib versus everolimus in advanced renal cell carcinoma (METEOR): final results from a randomised, open-label, phase 3 trial. Lancet Oncol. (2016) 17:917–27. 10.1016/S1470-2045(16)30107-3 27279544

[28] Motzer RJ Hutson TE Tomczak P Michaelson MD Bukowski RM Oudard S. Overall survival and updated results for sunitinib compared with interferon alfa in patients with metastatic renal cell carcinoma. J Clin Oncol. (2009) 27:3584–90. 10.1200/JCO.2008.20.1293 19487381 PMC3646307

[29] Sternberg CN Davis ID Mardiak J Szczylik C Lee E Wagstaff J Pazopanib in locally advanced or metastatic renal cell carcinoma: results of a randomized phase III trial. J Clin Oncol. (2010) 28:1061–8. 10.1200/JCO.2009.23.9764 20100962

[30] Sternberg CN Hawkins RE Wagstaff J Salman P Mardiak J Barrios CH A randomised, double-blind phase III study of pazopanib in patients with advanced and/or metastatic renal cell carcinoma: final overall survival results and safety update. Eur J Cancer. (2013) 49:1287–96. 10.1016/j.ejca.2012.12.010 23321547

[31] Cavaliere C D'Aniello C Pepa CD Pisconti S Berretta M Facchini G Current and emerging treatments for metastatic renal cell carcinoma. Curr Cancer Drug Targets. (2017) 8:468–79. 10.2174/1568009617666170209094030 28183256

[32] Vasudev NS Selby PJ Banks RE. Renal cancer biomarkers: the promise of personalized care. BMC Med. (2012) 10:112. 10.1186/1741-7015-10-112 23016578 PMC3521191

[33] Deng H Liu W He T Hong Z Yi F Wei Y. Comparative efficacy, safety, and costs of sorafenib vs. sunitinib as first-line therapy for metastatic renal cell carcinoma: a systematic review and meta-analysis. Front Oncol. (2019) 9:479. 10.3389/fonc.2019.00479 31293962 PMC6598399

[34] Sandock DS Seftel AD Resnick MI A new protocol for the follow-up of renal cell carcinoma based on pathological. J Urol. (1995) 154:28–31. 10.1016/S0022-5347(01)67215-X 7776446

[35] Sun M Shariat SF Cheng C Ficarra V Murai M Oudard S. Prognostic factors and predictive models in renal cell carcinoma: a contemporary review. Eur Urol. (2011) 60:644–61. 10.1016/j.eururo.2011.06.041 21741163

[36] Cavaliere C D'Aniello C Cecere S Di Napoli M Berretta M De Domenico R Renal cancer: prognostic and predictive biomarkers. In: Islam R editors. Prognostic and Predictive Response Therapy Factors in Cancer Disease (Colorectal, Breast, Liver, Lung, Gastric, Renal and Prostate Cancers). New York, NY: Nova Science Publishers (2015). p. 147–174.

[37] Morgia G Russo GI Do exist the “perfect” biomarker in metastatic renal cancer? WCRJ. (2014) 1:e415.

[38] Maitland ML Xu CF Cheng YC Kistner-Griffin E Ryan KA Karrison TG. Identification of a variant in KDR associated with serum VEGFR2 and pharmacodynamics of Pazopanib. Clin Cancer Res. (2015) 21:365–72. 10.1158/1078-0432.CCR-14-1683 25411163 PMC4323272

[39] D'Aniello C Cavaliere C Licchetta A Gnoni A Pisconti S Facchini G Metastatic renal cancer: prognostic and predictive biomarkers review. WCRJ. (2014) 1:e289.

[40] Choueiri TK Regan MM Rosenberg JE Oh WK Clement J Amato AM. Carbonic anhydrase IX and pathological features as predictors of outcome in patients with metastatic clear-cell renal cell carcinoma receiving vascular endothelial growth factor-targeted therapy. Br J Urol Int. (2010) 106:772–8. 10.1111/j.1464-410X.2010.09218.x 20230385

[41] D' Alterio C Portella L Ottaiano A Rizzo M Carteni G Pignata S High CXCR4 expression correlates with sunitinib poor response in metastatic renal cancer. Curr Can Drug Targets. (2012) 12:693–702. 10.2174/156800912801784820 22463589

[42] Guo JB Tang XN Sheng CL Use of CXCR4 expression to predict the efficacy of sorafenib treatment in patients with metastatic renal cell carcinoma. J Clin Oncol. (2011) 29:359 10.1200/jco.2011.29.7_suppl.359

[43] Motzer RJ Hutson TE Hudes GR Figlin RA Martini JF English PA. Investigation of novel circulating proteins, germ line single-nucleotide polymorphisms, and molecular tumor markers as potential efficacy biomarkers of first-line sunitinib therapy for advanced renal cell carcinoma. Cancer Chemother Pharmacol. (2014) 74:739–50. 10.1007/s00280-014-2539-0 25100134 PMC4175044

[44] López C Esteban E Astudillo A Pardo P Berros JP Izquierdo M Predictive factors for response to treatment in patients with advanced M renal cell carcinoma. Invest New Drugs. (2012) 30:2443–9. 10.1007/s10637-012-9836-4 22644070

[45] Bergantino F Guariniello S Raucci R Colonna G De Luca A Normanno N. Structure-fluctuation-function relationships of seven pro-angiogenic isoforms of VEGFA, important mediators of tumorigenesis. Biochim Biophys Acta. (2015) 1854:410–25. 10.1016/j.bbapap.2015.01.005 25617660

[46] Roskoski R Jr. VEGF receptor protein-tyrosine kinases: structure and regulation. Biochem Biophys Res Commun. (2008) 375:287–91. 10.1016/j.bbrc.2008.07.121 18680722

[47] Bowler E Oltean S. Alternative Splicing in Angiogenesis. Int J Mol Sci. (2019) 20:2067. 10.3390/ijms20092067 31027366 PMC6540211

[48] Cho WG Kleinman ME Dridi S Takeda A Baffi JZ Yamada K. Alternatively spliced vascular endothelial growth factor receptor-2 is an essential endogenous inhibitor of lymphatic vessel growth. Nat Med. (2009) 15:1023–30. 10.1038/nm.2018 19668192 PMC2882165

[49] Escudier B Eisen T Stadler WM Szczylik C Oudard S Staehler M. Sorafenib for treatment of renal cell carcinoma: final efficacy and safety results of the phase III treatment approaches in renal cancer global evaluation trial. J Clin Oncol. (2009) 27:3312–8. 10.1200/JCO.2008.19.5511 19451442

[50] Deprimo SE Bello CL Smeraglia J Baum CM Spinella D Rini BI. Circulating protein biomarkers of pharmacodynamic activity of sunitinib in patients with metastatic renal cell carcinoma: modulation of VEGF and VEGF-related proteins. J Transl Med. (2007) 5:32. 10.1186/1479-5876-5-32 17605814 PMC1939830

[51] Rini BI Michaelson MD Rosenberg JE Sosman JA Stadler WM Hutson TE. Antitumor activity and biomarker analysis of sunitinib in patients with bevacizumab-refractory metastatic renal cell carcinoma. J Clin Oncol. (2008) 26:3743–8. 10.1200/JCO.2007.15.5416 18669461

[52] Koo AS Armstrong C Bochner B Shimabukuro T Tso CL deKernion JB. Interleukin-6 and renal cell cancer: production, regulation, and growth effects. Cancer Immunol Immunother. (1992) 35:97. 10.1007/BF01741856 1596939 PMC11037957

[53] Tran HT Liu Y Zurita AJ Lin Y Baker-Neblett KL Martin AM Prognostic or predictive plasma cytokines and angiogenic factors for patients treated with pazopanib for metastatic renal-cell cancer: a retrospective analysis of phase 2 and phase 3 trials. Lancet Oncol. (2012) 13:827–37. 10.1016/S1470-2045(12)70241-3 22759480

[54] Zurita AJ Gagnon RC Liu Y Tran HT Figlin RA Hutson TE. Integrating cytokines and angiogenic factors and tumour bulk with selected clinical criteria improves determination of prognosis in advanced renal cell carcinoma. Br J Cancer. (2017) 117:478–84. 10.1038/bjc.2017.206 28683470 PMC5558688

[55] Apellániz-Ruiz M Diekstra MH Roldán JM Boven E Castellano D Gelderblom H. Evaluation of KDR rs34231037 as a predictor of sunitinib efficacy in patients with metastatic renal cell carcinoma. Pharmacogenet Genomics. (2017) 27:227–31. 10.1097/FPC.0000000000000280 28430711

[56] Sanford T Chung PH Reinish A Valera V Srinivasan R Linehan WM. Molecular sub-classification of renal epithelial tumors using meta-analysis of gene expression microarrays. PLoS ONE. (2011) 6:e21260. 10.1371/journal.pone.0021260 21818257 PMC3144884

[57] Eisengart LJ MacVicar GR Yang XJ. Predictors of response to targeted therapy in renal cell carcinoma. Arch Pathol Lab. (2012) 136:490–5. 10.5858/arpa.2010-0308-RA 22229848

[58] Kim BY Kim H Cho EJ Youn HD. Nur77 upregulates HIF-alpha by inhibiting pVHL-mediated degradation. Exp Mol. (2008) 40:71–83. 10.3858/emm.2008.40.1.71 18305400 PMC2679322

[59] Choueiri TK Vaziri SA Jaeger E Elson P Wood L Bhalla IP. Von Hippel-Lindau gene status and response to vascular endothelial growth factor targeted therapy for metastatic clear cell renal cell carcinoma. J Urol. (2008) 180:860–6. 10.1016/j.juro.2008.05.015 18635227

[60] Choueiri TK Fay AP Gagnon R Lin Y Bahamon B Brown V. The role of aberrant VHL/HIF pathway elements in predicting clinical outcome to pazopanib therapy in patients with metastatic clear-cell renal cell carcinoma. Clin Cancer. (2013) 19:5218–26. 10.1158/1078-0432.CCR-13-0491 23881929 PMC4522695

[61] Xu K Liu P Wei W. mTOR signaling in tumorigenesis. Biochim Biophys Acta. (2014) 1846:638–54. 10.1016/j.bbcan.2014.10.007 25450580 PMC4261029

[62] Peña C Lathia C Shan M Escudier B Bukowski RM. Biomarkers predicting outcome in patients with advanced renal cell carcinoma: results from sorafenib phase III Treatment Approaches in Renal Cancer Global Evaluation Trial. Clin Cancer. (2010) 16:4853–63. 10.1158/1078-0432.CCR-09-3343 20651059

[63] Varela I Tarpey P Raine K Huang D Ong CK Stephens P. Exome sequencing identifies frequent mutation of the SWI/SNF complex gene PBRM1 in renal carcinoma. Nature. (2011) 469:539–42. 10.1038/nature09639 21248752 PMC3030920

[64] Young AC Craven RA Cohen D Taylor C Booth C Harnden P. Analysis of VHL gene alterations and their relationship to clinical parameters in sporadic conventional renal cell carcinoma. Clin Cancer. (2009) 15:7582−92. 10.1158/1078-0432.CCR-09-2131 19996202 PMC2795746

[65] Nickerson ML Jaeger E Shi Y Durocher JA Mahurkar S Zaridze D. Improved identification of von Hippel-Lindau gene alterations in clear cell renal tumors. Clin Cancer. (2008) 14:4726–34. 10.1158/1078-0432.CCR-07-4921 18676741 PMC2629664

[66] Peña-Llopis S Vega-Rubín-de-Celis S Liao A Leng N Pavía-Jiménez A Wang S. BAP1 loss defines a new class of renal cell carcinoma. Nat Genet. (2012) 44:751–9. 10.1038/ng.2323 22683710 PMC3788680

[67] Kapur P Peña-Llopis S Christie A Zhrebker L Pavía-Jiménez A Rathmell WK. Effects on survival of BAP1 and PBRM1 mutations in sporadic clear-cell renal-cell carcinoma: a retrospective analysis with independent validation. Lancet Oncol. (2013) 14:159–67. 10.1016/S1470-2045(12)70584-3 23333114 PMC4674067

[68] Gossage L Murtaza M Slatter AF Lichtenstein CP Warren A Haynes B. Clinical and pathological impact of VHL, PBRM1, BAP1, SETD2, KDM6A, and JARID1c in clear cell renal cell carcinoma. Genes Chromosomes Cancer. (2014) 53:38–51. 10.1002/gcc.22116 24166983

[69] Dalgliesh GL Furge K Greenman C Chen L Bignell G Butler A. Systematic sequencing of renal carcinoma reveals inactivation of histone modifying genes. Nature. (2010) 463:360–3. 10.1038/nature08672 20054297 PMC2820242

[70] Purdue MP Johansson M Zelenika D Toro JR Scelo G Moore LE Genome-wide association study of renal cell carcinoma identifies two susceptibility loci on 2p21 q13.3. Genome-wide association study of renal cell carcinoma identifies two susceptibility loci on 2p21 q13.3. Nat Genet. (2011) 43:60–5. 10.1038/ng.723 21131975 PMC3049257

[71] Wu X Scelo G Purdue MP Rothman N Johansson M Ye Y. A genome-wide association study identifies a novel susceptibility locus for renal cell carcinoma on 12p11.23. Hum Mol Genet. (2012) 21:456–62. 10.1093/hmg/ddr479 22010048 PMC3276284

[72] Xu CF Bing NX Ball HA Sternberg CN Hutson TE de Souza P. Pazopanib efficacy in renal cell carcinoma: evidence for predictive genetic markers in angiogenesis-related and exposure-related genes. J Clin Oncol. (2011) 29:2557–64. 10.1200/JCO.2010.32.9110 21576632

[73] Huang D Ding Y Zhou M Rini BI Petillo D Qian CN. Interleukin-8 mediates resistance toantiangiogenic agent sunitinib in renal cell carcinoma. Cancer. (2010) 70:1063–71. 10.1158/0008-5472.CAN-09-3965 20103651 PMC3719378

[74] Xu CF Johnson T Garcia-Donas J Choueiri TK Sternberg CN Davis ID IL8 polymorphisms and overall survival in pazopanib- or sunitinib-treated patients with renal cell carcinoma. Br J Cancer. (2015) 112:1190–8. 10.1038/bjc.2015.64 25695485 PMC4385958

[75] Garcia-Donas J Esteban E Leandro-Garcia LJ Castellano DE González del Alba A Climent MA. Single nucleotide polymorphism associations with response and toxic effects in patients with advanced renal-cell carcinoma treated with first-line sunitinib: a multicentre, observational, prospective study. Lancet Oncol. (2011) 12:1143–50. 10.1016/S1470-2045(11)70266-2 22015057

[76] Kim JJ Vaziri SA Rini BI Elson P Garcia JA Wirka R. Association of VEGF and VEGFR2 single nucleotide polymorphisms with hypertension and clinical outcome in metastatic clear cell renal cell carcinoma patients treated with sunitinib. Cancer. (2012) 118:1946–54. 10.1002/cncr.26491 21882181 PMC4124607

[77] van der Veldt AA Eechoute K Gelderblom H Gietema J Guchelaar HJ van Erp NP. Genetic polymorphisms associated with a prolonged progression-free survival in patients with metastatic renal cell cancer treated with sunitinib. Clin Cancer. (2010) 11:17:620–9. 10.1158/1078-0432.CCR-10-1828 21097692

[78] Escudier B Rini BI Motzer RJ Tarazi J Kim S Huang X. Genotype correlations with blood pressure and efficacy from a randomized phase III trial of second-line axitinib versus sorafenib in metastatic renal cell carcinoma. Clin Genitourin Cancer. (2015) 13:328–37. 10.1016/j.clgc.2015.02.007 25816720

[79] Escudier B Loomis AK Kaprin A Motzer R Tomczak P Tarazi J Association of single nucleotide polymorphisms (SNPs) in VEGF pathway genes with progression-free survival (PFS) and blood pressure in metastatic renal cell carcinoma (mRCC) in the phase 3 trial of axitinib vs. sorafenib (AXIS trial). In european multidisciplinary cancer congress on integrating basic and translational science, surgery, radiotherapy, medical oncology, advocacy and care. Eur J Cancer. (2011) 47:S505 10.1016/S0959-8049(11)72018-4

[80] Liu X Swen JJ Boven E Castellano D Gelderblom H Mathijssen RH Rodríguez-Antona C9,10, García-Donas J6,11, Rini BI12, Guchelaar HJ1Meta-analysis on the association of VEGFR1 genetic variants with sunitinib outcome in metastatic renal cell carcinoma patients. Oncotarget. (2017) 8:1204–12. 10.18632/oncotarget.13597 27901483 PMC5352048

[81] Badal SS Danesh FR. MicroRNAs and their applications in kidney diseases. Pediatr Nephrol. (2015) 30:727–40. 10.1007/s00467-014-2867-7 24928414 PMC4265577

[82] McCormick RI Blick C Ragoussis J Schoedel J Mole DR Young AC. miR-210 is a target of hypoxia-inducible factors 1 and 2 in renal cancer, regulates ISCU and correlates with good prognosis. Br J Cancer. (2013) 108:1133–42. 10.1038/bjc.2013.56 23449350 PMC3619073

[83] Neal CS Michael MZ Rawlings LH Van der Hoek MB Gleadle JM. The VHL dependent regulation of microRNAs in renal cancer. BMC Med. (2010) 8:64. 10.1186/1741-7015-8-64 20964835 PMC2978113

[84] Samaan S Khella HW Girgis A Scorilas A Lianidou E Gabril M. miR-210 is a prognostic marker in clear cell renal cell carcinoma. J Mol Diagn. (2015) 17:136–44. 10.1016/j.jmoldx.2014.10.005 25555365

[85] Khella HW Scorilas A Mozes R Mirham L Lianidou E Krylov SN Low expression of miR126 is a prognostic marker for metastatic clear cell renal cell carcinoma. Am J Pathol. (2015) 185:693–703. 10.1016/j.ajpath.2014.11.017 25572155

[86] Gowrishankar B Ibragimova I Zhou Y Slifker MJ Devarajan K Al-Saleem T. MicroRNA expression signatures of stage, grade, and progression in clear cell RCC. Cancer Biol Ther. (2014) 15:329–41. 10.4161/cbt.27314 24351440 PMC3974834

[87] Prior C Perez-Gracia JL Garcia-Donas J Rodriguez-Antona C Guruceaga E Esteban E. Identification of tissue microRNAs predictive of sunitinib activity in patients with metastatic renal cell carcinoma. PLoS ONE. (2014) 9:e86263. 10.1371/journal.pone.0086263 24475095 PMC3901669

[88] Berkers J Govaere O Wolter P Beuselinck B Schöffski P van Kempen LC. A possible role for microRNA-141 down-regulation in sunitinib resistant metastatic clear cell renal cell carcinoma through induction of epithelialto-mesenchymal transition and hypoxia resistance. J Urol. (2013) 189:1930–8. 10.1016/j.juro.2012.11.133 23206420

[89] Chen F Zhang Y Senbabaoglu Y Ciriello G Yang L Reznik E. Multilevel genomics-based taxonomy of renal cell carcinoma. Cell Rep. (2016) 14:2476–89. 10.1016/j.celrep.2016.02.024 26947078 PMC4794376

[90] Brannon AR Reddy A Seiler M Arreola A Moore DT Pruthi RS Molecular stratification of clear cell renal cell carcinoma by consensus clustering reveals distinct subtypes and survival patterns. Genes Cancer. (2010) 1:152–63. 10.1177/1947601909359929 20871783 PMC2943630

[91] Brannon AR Haake SM Hacker KE Pruthi RS Wallen EM Nielsen ME. Meta-analysis of clear cell renal cell carcinoma gene expression defines a variant subgroup and identifies gender influences on tumor biology. Eur Urol. (2012) 61:258–68. 10.1016/j.eururo.2011.10.007 22030119 PMC3244546

[92] Brooks SA Brannon AR Parker JS Fisher JC Sen O Kattan MW ClearCode34: a prognostic risk predictor for localized clear cell renal cell carcinoma. Eur Urol. (2014) 66:77–84. 10.1016/j.eururo.2014.02.035 24613583 PMC4058355

[93] Cancer Genome Atlas Research Network Comprehensive molecular characterization of clear cell renal cell carcinoma. Nature. (2013) 499:43–9. 10.1038/nature12222 23792563 PMC3771322

[94] Hakimi AA Reznik E Lee CH Creighton CJ Brannon AR Luna A. An Integrated Metabolic Atlas of Clear Cell Renal Cell Carcinoma. Cancer Cell. (2016) 29:104–16. 10.1016/j.ccell.2015.12.004 26766592 PMC4809063

[95] de Velasco G Culhane AC Fay AP Hakimi AA Voss MH Tannir NM. Molecular subtypes improve prognostic value of international metastatic renal cell carcinoma database consortium prognostic model. Oncologist. (2017) 22:286–92. 10.1634/theoncologist.2016-0078 28220024 PMC5344647

[96] Beuselinck B Job S Becht E Karadimou A Verkarre V Couchy G. Molecular subtypes of clear cell renal cell carcinoma are associated with sunitinib response in the metastatic setting. Clin Cancer Res. (2015) 21:1329–39. 10.1158/1078-0432.CCR-14-1128 25583177

[97] Beuselinck B Verbiest A Couchy G Job S de Reynies A Meiller C. Pro-angiogenic gene expression is associated with better outcome on sunitinib in metastatic clear-cell renal cell carcinoma. Acta Oncol. (2018) 57:498–508. 10.1080/0284186X.2017.1388927 29095068

[98] Verbiest A Couchy G Job S Zucman-Rossi J Caruana L Lerut E. Molecular subtypes of clear cell renal cell carcinoma are associated with outcome during pazopanib therapy in the metastatic setting. Clin Genitourin Cancer. (2018) 16:e605–12. 10.1016/j.clgc.2017.10.017 29239846

[99] Verbiest A Couchy G Job S Caruana L Lerut E Oyen R. Molecular subtypes of clear-cell renal cell carcinoma are prognostic for outcome after complete metastasectomy. Eur Urol. (2018) 74:474–80. 10.1016/j.eururo.2018.01.042 29463434

[100] Voss MH Kuo F Chen D Marker M Patel P Redzematovic A Integrated biomarker analysis 2 renal cell cancer (RCC) patients (pts) treated on the phase 3 COMPARZ trial: correlating common mutation events in PBRM1 and BAP1 with angiogenesis expression signatures and outcomes on tyrosine kinase inhibitor (TKI) therapy. J Clin Oncol. (2017) 35 (15_suppl):4523 10.1200/JCO.2017.35.15_suppl.4523

[101] Verbiest A Renders I Caruso S Couchy G Job S Laenen A. Clear-cell renal cell carcinoma: molecular characterization of IMDC risk groups and sarcomatoid tumors. Clin Genitourin Cancer. (2019) 17:e981–94. 10.1016/j.clgc.2019.05.009 31229459

[102] Choueiri TK Fay AP Gray KP Callea M Ho TH Albiges L. PD-L1 expression in nonclear-cell renal cell carcinoma. Ann Oncol. (2014) 25:2178–84. 10.1093/annonc/mdu445 25193987 PMC4288138

[103] Thompson RH Dong H Lohse CM Leibovich BC Blute ML Cheville JC. PD-1 is expressed by tumor-infiltrating immune cells and is associated with poor outcome for patients with renal cell carcinoma. Clin Cancer Res. (2007) 13:1757–61 10.1158/1078-0432.CCR-06-2599 17363529

[104] Motzer RJ Escudier B McDermott DF George S Hammers HJ Srinivas S. Nivolumab versus Everolimus in Advanced Renal-Cell Carcinoma. N Engl J Med. (2015) 373:1803–13. 10.1056/NEJMoa1510665 26406148 PMC5719487

[105] Brauer MJ Zhuang G Schmidt M Yao J Wu X Kaminker JS. Identification and analysis of *in vivo* VEGF downstream markers link VEGF pathway activity with efficacy of anti-VEGF therapies. Clin Cancer Res. (2013) 19:3681–92. 10.1158/1078-0432.CCR-12-3635 23685835

[106] Lopez-Beltran A Henriques V Cimadamore A Santoni M Cheng L Gevaert T. The identification of immunological biomarkers in kidney cancers. Front Oncol. (2018) 8:456. 10.3389/fonc.2018.00456 30450335 PMC6225533

[107] Motzer RJ Tannir NM McDermott DF Arén Frontera O Melichar B Choueiri TK. Nivolumab plus ipilimumab versus sunitinib in advanced renal-cell carcinoma. N Engl J Med. (2018) 378:1277–90. 10.1056/NEJMoa1712126 29562145 PMC5972549

[108] Hakimi AA Voss MH Kuo F Sanchez A Liu M Nixon BG. Transcriptomic profiling of the tumor microenvironment reveals distinct subgroups of clear cell renal cell cancer: data from a randomized phase III trial. Cancer Discov. (2019) 9:510–25. 10.1158/2159-8290.CD-18-0957 30622105 PMC6697163

[109] Motzer RJ Penkov K Haanen J Rini B Albiges L Campbell MT. Avelumab plus axitinib versus sunitinib for advanced renal-cell carcinoma. N Engl J Med. (2019) 380:1103–15. 10.1056/NEJMoa1816047 30779531 PMC6716603

[110] Rini BI Powles T Atkins MB Escudier B McDermott DF Suarez C. Atezolizumab plus bevacizumab versus sunitinib in patients with previously untreated metastatic renal cell carcinoma (IMmotion151): a multicentre, open-label, phase 3, randomised controlled trial. Lancet. (2019) 393:2404–15. 10.1016/S0140-6736(19)30723-8 31079938

[111] Rini BI Plimack ER Stus V Gafanov R Hawkins R Nosov D. Pembrolizumab plus axitinib versus sunitinib for advanced renal-cell carcinoma. N Engl J Med. (2019) 380:1116–27. 10.1056/NEJMoa1816714 30779529

[112] McDermott DF Huseni MA Atkins MB Motzer RJ Rini BI Escudier B Clinical activity and molecular correlates of response to atezolizumab alone or in combination with bevacizumab versus sunitinib in renal cell carcinoma. Nat Med. (2018) 24:749–57. 10.1038/s41591-018-0053-3 29867230 PMC6721896

[113] Gerlinger M Horswell S Larkin J Rowan AJ Salm MP Varela I. Genomic architecture and evolution of clear cell renal cell carcinomas defined by multiregion sequencing. Nat Genet. (2014) 46:225–33. 10.1038/ng.2891 24487277 PMC4636053

[114] Mitchell TJ Rossi SH Klatte T Stewart GD. Genomics and clinical correlates of renal cell carcinoma. World J Urol. (2018) 36:1899–911. 10.1007/s00345-018-2429-x 30099580 PMC6280817

[115] Serie DJ Joseph RW Cheville JC Ho TH Parasramka M Hilton T. Clear cell type A and B molecular subtypes in metastatic clear cell renal cell carcinoma: tumor heterogeneity and aggressiveness. Eur Urol. (2017) 71:979–85. 10.1016/j.eururo.2016.11.018 27899233 PMC5401797

[116] Tomita Y Shinohara N Yuasa T Fujimoto H Niwakawa M Mugiya S. Overall survival and updated results from a phase II study of sunitinib in Japanese patients with metastatic renal cell carcinoma. Jpn J Clin Oncol. (2010) 40:1166–72. 10.1093/jjco/hyq146 20713418

[117] Gore ME Szczylik C Porta C Bracarda S Bjarnason GA Oudard S. Safety and efficacy of sunitinib for metastatic renal-cell carcinoma: an expanded-access trial. Lancet Oncol. (2009) 10:757–63. 10.1016/S1470-2045(09)70162-7 19615940

[118] Veronese ML Mosenkis A Flaherty KT Gallagher M Stevenson JP Townsend RR. Mechanisms of hypertension associated with −9006. J Clin Oncol. (2006) 24:1363–9. 10.1200/JCO.2005.02.0503 16446323

[119] Bono P Elfving H Utriainen T Osterlund P Saarto T Alanko T. Hypertension and clinical benefit of bevacizumab in the treatment of advanced renal cell carcinoma. Ann Oncol. (2009) 20:393–4. 10.1093/annonc/mdn729 19211503

[120] D'Aniello C Vitale MG Farnesi A Calvetti L Laterza MM Cavaliere C. Axitinib after sunitinib in metastatic renal cancer: preliminary results from italian “real world” SAX study. Front Pharmacol. (2016) 7:331. 10.3389/fphar.2016.00331 27733829 PMC5039205

[121] Cecere SC Rossetti S Cavaliere C Della Pepa C Di Napoli M Crispo A Pazopanib in metastatic renal cancer: a “Real-World” experience at national cancer institute “Fondazione G. Pascale. Front Pharmacol. (2016) 7:287 10.3389/fphar.2016.00287 27630568 PMC5005368

[122] Rini BI Tamaskar I Shaheen P Wood L Reddy S Dreicer R. Hypothyroidism in patients with metastatic renal cell carcinoma treated with sunitinib. J Natl Cancer Inst. (2007) 99:81–3. 10.1093/jnci/djk008 17202116

[123] Wolter P Dumez H Schoffski P. Sunitinib and hypothyroidism. N Engl J. (2007) 356:1580–81. 10.1056/NEJMc070327 17429091

[124] Wong E Rosen LS Mulay M Vanvugt A Dinolfo M Tomoda C. Sunitinib induces hypothyroidism in advanced cancer patients and may inhibit thyroid peroxidase activity. Thyroid. (2007) 17:351–5. 10.1089/thy.2006.0308 17465866

[125] Baffert F Le T Thurston G McDonald DM. Angiopoietin-1 decreases plasma leakage by reducing number and size of endothelial gaps in venules. Am J Physiol Heart Circ Physiol. (2006) 290:H107–18. 10.1152/ajpheart.00542.2005 16126815

[126] Grossmann M Premaratne E Desai J Davis ID. Thyrotoxicosis during sunitinib treatment for renal cell carcinoma. Clin Endocrinol. (2008) 69:669–72. 10.1111/j.1365-2265.2008.03253.x 18394019

[127] Mannavola D Coco P Vannucchi G Bertuelli R Carletto M Casali PG. A novel tyrosine-kinase selective inhibitor, sunitinib, induces transient hypothyroidism by blocking iodine uptake. J Clin Endocrinol Metab. (2007) 92:3531–4. 10.1210/jc.2007-0586 17595247

[128] Wolter P Stephan C Decallonne B Evaluation of thyroid dysfunction as a candidate surrogate marker for efficacy of sunitinib in patients (pts) with advanced renal cell cancer (RCC). J Clin Oncol. (2008) 26 (Suppl.):5126 10.1200/jco.2008.26.15_suppl.512618838707

[129] Schmidinger M Vogl UM Bojic M Lamm W Heinzl H Haitel A Hypothyroidism in patients with renal cell carcinoma: blessing or curse? Cancer. (2011) 117:534–44. 10.1002/cncr.25422 20845482

[130] Pinto A Moreno V Aguayo C Hypothyroidism and macrocytosis as surrogate markers for response and survival in patients with advanced renal cell carcinoma treatment with sunitinib as first-line therapy. EJC. (2011) 47:S521 10.1016/S0959-8049(11)72069-X

[131] Sabatier R Eymard JC Walz J Deville JL Narbonne H Boher JM. Could thyroid dysfunction influence outcome in sunitinib-treated metastatic renal cell carcinoma? Ann Oncol. (2012) 23:714–21. 10.1093/annonc/mdr275 21653681

[132] Nearchou A Valachis A Lind P Akre O Sandström P. Acquired hypothyroidism as a predictive marker of outcome in patients with metastatic renal cell carcinoma treated with tyrosine kinase inhibitors: a literature-based meta-analysis. Clin Genitourin Cancer. (2015) 13:280–6. 10.1016/j.clgc.2014.10.002 25442773

[133] Lacouture ME Reilly LM Gerami P Guitart J. Hand foot skin reaction in cancer patients treated with the multikinase inhibitors sorafenib and sunitinib. Ann Oncol. (2008) 19:1955–61. 10.1093/annonc/mdn389 18550575

[134] Erber R Thurnher A Katsen AD Groth G Kerger H Hammes HP. Combined inhibition of VEGF and PDGF signaling enforces tumor vessel regression by interfering with pericyte-mediated endothelial cell survival mechanisms. FASEB J. (2004) 18:338–40. 10.1096/fj.03-0271fje 14657001

[135] Yang CH Lin WC Chuang CK Chang YC Pang ST Lin YC. Hand-foot skin reaction in patients treated with sorafenib: a clinicopathological study of cutaneous manifestations due to multitargeted kinase inhibitor therapy. Br J Dermatol. (2008) 158:592–6. 10.1111/j.1365-2133.2007.08357.x 18070211

[136] Pusanov I Michaelson D Cohen D Evaluation of hand-foot syndrome (HFS) as a potential biomarker of sunitinib efficacy in patients with metastatic renal cell carcinoma (mRCC) and gastrointestinal stromal tumour (GIST). J Clin Oncol. (2011) 29(15_suppl):e21113 10.1200/jco.2011.29.15_suppl.e21113

[137] Davis M Figlin R Hutson TE Asthenia and fatigue as potential biomarkers of sunitinib efficacy in metastatic renal cell carcinoma. EJC. (2011) 47:S135 10.1016/S0959-8049(11)70782-1

[138] Donskov F Michaelson MD Puzanov I Comparative assessment of sunitinib associated adverse events as potential biomarkers of efficacy in metastatic renal cell carcinoma (mRCC). Ann Oncol. (2012) 23(Suppl. 9).

[139] Larkin JM Pyle LM Gore ME. Fatigue in renal cell carcinoma: the hidden burden of current targeted therapies. Oncologist. (2010) 15:1135–46. 10.1634/theoncologist.2010-0078 21051659 PMC3227914

[140] Wolter P Wildiers D Vanderschueren H Hypogonadism in male patients treated with the tyrosine kinase inhibitors sunitinib or sorafenib. J Clin Oncol. (2009) 27 (Suppl. 15s):3565.

[141] Dudani S Graham J Wells JC Bakouny Z Pal SK Dizman N. First-line immuno-oncology combination therapies in metastatic renal-cell carcinoma: results from the international metastatic renal-cell carcinoma database consortium. Eur Urol. (2019) 76:861–7. 10.1016/j.eururo.2019.07.048 31445844 PMC6858928

[142] Hahn AW Klaassen Z Agarwal N Haaland B Esther J Ye XY. First-line treatment of metastatic renal cell carcinoma: a systematic review and network meta-analysis. Eur Urol Oncol. (2019) 2:708–15. 10.1016/j.euo.2019.09.002 31588018

[143] Al-Marrawi MY Rini BI Harshman LC Bjarnason G Wood L Vaishampayan U. The association of clinical outcome to first-line VEGF-targeted therapy with clinical outcome to second-line VEGFtargeted therapy in metastatic renal cell carcinoma patients. Target Oncol. (2013) 8:203–9. 10.1007/s11523-012-0252-7 23300029 PMC4144038

[144] Elaidi R Harbaoui A Beuselinck B Eymard JC Bamias A DeGuillebon E. Outcomes from second-line therapyin long-term responders to first-line tyrosine kinase inhibitor in clear-cell metastatic renal cell carcinoma. Ann Oncol. (2015) 26:378–85. 10.1093/annonc/mdu552 25467013

[145] Iacovelli R Cartenì G Sternberg CN Milella M Santoni M Di Lorenzo G. Clinical outcomes in patients receiving three lines of targeted therapy for metastatic renal cell carcinoma: results from a large patient cohort. Eur J Cancer. (2013) 49:2134–42. 10.1016/j.ejca.2013.02.032 23518211

[146] Seidel C Busch J Weikert S Steffens S Fenner M Ganser A. Progression free survival of first line vascular endothelial growth factor-targeted therapy is an important prognostic parameter in patients with metastatic renal cell carcinoma. Eur J Cancer. (2012) 48:1023–30. 10.1016/j.ejca.2012.02.048 22436979

[147] Iacovelli R Lanoy E Albiges L Escudier B. Tumour burden is an independent prognostic factor in metastatic renal cell carcinoma. BJU Int. (2012) 110:1747–53. 10.1111/j.1464-410X.2012.11518.x 23106948

[148] Basappa NS Elson P Golshayan AR Wood L Garcia JA Dreicer R. The impact of tumor burden characteristics in patients with metastatic renal cell carcinoma treated with sunitinib. Cancer. (2011) 117:1183–9. 10.1002/cncr.25713 20960527

[149] Escudier B Michaelson MD Motzer RJ Hutson TE Clark JI Lim HY. Axitinib versus sorafenib in advanced renal cell carcinoma: subanalyses by prior therapy from a randomised phase III trial. Br J Cancer. (2014) 110:2821–8. 10.1038/bjc.2014.244 24823696 PMC4056058

[150] Calvo E Escudier B Motzer RJ Oudard S Hutson TE Porta C Everolimus in metastatic renal cell carcinoma: subgroup analysis of patients with 1 or 2 previous vascular endothelial growth factor receptor-tyrosine kinase inhibitor therapies enrolled in the phase III RECORD-1 study. Eur J Cancer. (2012) 48:333–9. 10.1016/j.ejca.2011.11.027 22209391

[151] Bex A Larkin J Voss M Challenging the treatment paradigm for advanced renal cell carcinoma: a review of systemic and localized therapies. Am Soc Clin Oncol Educ Book. (2015) 2015:e239–47. 10.14694/EdBook_AM.2015.35.e239 25993179

[152] Motzer RJ Rini BI McDermott DF Redman BG Kuzel TM Harrison MR Nivolumab for metastatic renal cell carcinoma (mRCC): results of a randomized, dose-ranging phase II trial. J Clin Oncol. (2015) 33:1430–7. 10.1200/JCO.2014.59.0703 25452452 PMC4806782

[153] Choueiri TK Escudier B Powles T Mainwaring PN Rini BI Donskov F. Cabozantinib versus everolimus in advanced renal-cell carcinoma. N Engl J Med. (2015) 373:1814–23. 10.1056/NEJMoa1510016 26406150 PMC5024539

[154] Escudier B Sharma P McDermott DF George S Hammers HJ Srinivas S. CheckMate 025 randomized phase 3 study: outcomes by key baseline factors and prior therapy for nivolumab versus everolimus in advanced renal cell carcinoma. Eur Urol. (2017) 72:962–71. 10.1016/j.eururo.2017.02.010 28262413

[155] Bracarda S Bamias A Casper J Negrier S Sella A Staehler M. Is Axitinib still a valid Option for mRCC in the second-line setting? Prognostic Factor Analyses from the AXIS Trial. Clin Genitourin Cancer. (2019) 17:e689–e703. 10.1016/j.clgc.2019.03.017 31072748

[156] Auvray M Auclin E Barthelemy P Bono P Kellokumpu-Lehtinen P Gross-Goupil M. Second-line targeted therapies after nivolumab-ipilimumab failure in metastatic renal cell carcinoma. Eur J Cancer. (2019) 108:33–40. 10.1016/j.ejca.2018.11.031 30616146

[157] Shah AY Kotecha RR Lemke EA Chandramohan A Chaim JL Msaouel P. Outcomes of patients with metastatic clear-cell renal cell carcinoma treated with second-line VEGFR-TKI after first-line immune checkpoint inhibitors. Eur J Cancer. (2019) 114:67–75. 10.1016/j.ejca.2019.04.003 31075726 PMC7537491

[158] Nadal R Amin A Geynisman DM Voss MH Weinstock M Doyle J. Safety and clinical activity of vascular endothelial growth factor receptor (VEGFR)-tyrosine kinase inhibitors after programmed cell death 1 inhibitor treatment in patients with metastatic clear cell renal cell carcinoma. Ann Oncol. (2016) 27:1304–11. 10.1093/annonc/mdw160 27059553 PMC6276905

